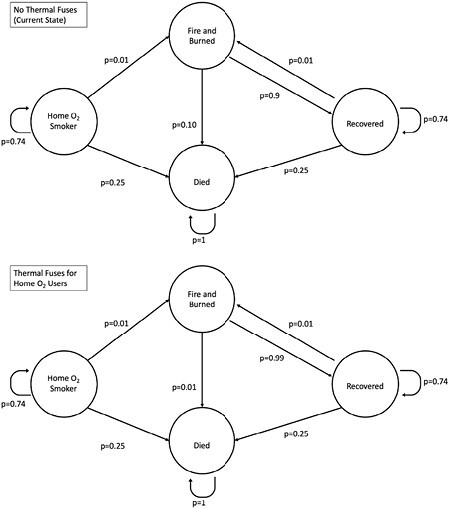# 52 Thermal Fuse Mandate Is Cost-effective for Home-oxygen Users in Prevention of Morbidity/mortality and Property Loss

**DOI:** 10.1093/jbcr/irae036.076

**Published:** 2024-04-17

**Authors:** Clifford C Sheckter, Rebecca Coffey

**Affiliations:** Stanford/Santa Clara Valley Medical Center, San Jose, CA; Parkland Health, Keller, TX; Stanford/Santa Clara Valley Medical Center, San Jose, CA; Parkland Health, Keller, TX

## Abstract

**Introduction:**

Smoking tobacco products while using home oxygen can lead to fires and explosions which cause cutaneous burns, death, and loss of property. Thermal fuses interrupt the propagation of ignited oxygen-lines and significantly reduce the risk of explosion and death. Mandating thermal fuses for all home oxygen users is costly due to the number of people affected and distribution of these resources. On the other hand, the cost of burn care and property loss may exceed the summed device cost. Prior to mandating thermal fuses for all home oxygen users in the US, cost-effectiveness analysis should be performed.

**Methods:**

A Markov model with annual cycling was constructed for the entire US population currently using home oxygen for chronic obstructive pulmonary disease (COPD). The probability of burn, death, and property loss were derived from a systematic review of literature. Societal and Medicare perspectives were adopted evaluating the costs of a federal policy including purchasing and shipping thermal fuses to all home oxygen users. Additional costs included the healthcare required to treat burn patients and the cost of prolonging these lives in advanced COPD. Cost savings included the avoided property loss in 2023 US dollars. Effectiveness was measured in gains in quality adjusted life years (QALYS) based on utilities derived from burn survivors and persons living with COPD. Deterministic and probabilistic sensitivity (10,000 Monte Carlo simulations) analysis was performed for key parameters including the cost of thermal fuses, mortality reduction from thermal fuses, and number of home oxygen fires per year. Discounting was 3% per year.

**Results:**

In the status quo, the 10-year societal cost was $28.67 billion compared to $28.36 billion in the policy mandate (saving $305.40 million at ten years). 1,812 QALYs were gained with the policy mandate, yielding and ICER of -$160,317. For the Medicare payor perspective, the incremental cost-effectiveness ratio (ICER) was $64,981. Deterministic and probabilistic sensitivity analyses showed little variation in the ICER under multiple scenarios. The discrepancy between the dominant ICER for societal perspective and cost-effective ICER for Medicare perspective reflected savings from averted property loss not realized by Medicare.

**Conclusions:**

A national policy mandating and paying for thermal fuses for all home oxygen users is dominant from a societal perspective and cost-effective from a Medicare perspective. The US federal government should adopt such a policy.

**Applicability of Research to Practice:**

This rigorous model demonstrates overwhelming support for a policy that mandates thermal fuses for home oxygen users including direct shipping the device to individuals. Federal and state governments should adopt this policy.